# Recognition of an Odour Pattern from *Paenibacillus larvae* Spore Samples by Trained Detection Dogs

**DOI:** 10.3390/ani13010154

**Published:** 2022-12-30

**Authors:** Neroli Thomson, Michelle Taylor, Pete Gifford, James Sainsbury, Sarah Cross

**Affiliations:** 1 School of Veterinary Science, Massey University, Palmerston North 4410, New Zealand; 2 The New Zealand Institute for Plant and Food Research Limited, Hamilton 3214, New Zealand; 3 K9 Search Medical Detections, Beaconsfield 4779, New Zealand

**Keywords:** working dogs, detection dogs, odour detection, American Foulbrood, spores, volatile organic compounds, *Paenibacillus larvae*, apiculture

## Abstract

**Simple Summary:**

American Foulbrood (AFB) is a disease of honey bee colonies caused by the spore-forming bacteria *Paenibacillus larvae*. Honey bee larvae become infected by consuming *P. larvae* spores in their food and subsequently die 9–12 days into development. The spores enter hives via bees taking honey from infected colonies or beekeepers inadvertently introducing *P. larvae* spores through contaminated hive ware, honey or pollen. In this study, we investigated whether detection dogs could be trained to recognise an odour pattern from *P. larvae* spores. The potential advantage of using dogs to detect *P. larvae* spore contamination is their ability to quickly work through large numbers of samples, such as working hives or empty brood boxes, facilitating more thorough inspections than would otherwise be possible. Positive indications could be checked with molecular testing and the items quarantined to prevent the further spread of AFB. However, as spores are metabolically inactive, it was unknown whether they would produce enough volatile compounds to be recognised by dogs. Therefore, the aim of this study was to determine whether detection dogs could recognise an odour pattern from *P. larvae* spores in a controlled setting, as proof of concept for future field trials. Three experienced detection dogs underwent target-odour training using a series of laboratory-produced spore and control samples. Two of the dogs successfully completed training and were then tested in a series of six trials in an odour-detection carousel. These trials were carefully designed to avoid inadvertently giving cues to the dogs. For instance, a new set of samples was used and the dog trainer was unaware of the location of the spore samples. Both dogs correctly identified the single spore sample from the seven control samples in all trials (100% success rate), demonstrating that detection dogs can indeed recognise an odour from *P. larvae* spore samples.

**Abstract:**

Spores of the bacteria *Paenibacillus larvae* play a central role in the transmission of American Foulbrood (AFB), a major disease of honey bee (*Apis mellifera*) colonies. This study investigated whether trained detection dogs could recognise an odour pattern from *P. larvae* spore samples. Although dogs have previously been used to detect diseased larvae in colonies with AFB, this is the first time they have been investigated for detecting *P. larvae* spore samples. Given that spores are metabolically inactive, it was unknown whether the spore samples would produce enough volatile organic compounds to form an odour pattern that could be detected by dogs. Three dogs were trained to identify laboratory-produced *P. larvae* spore samples and were systematically desensitized to non-target odours with a series of control samples. Two of the dogs successfully completed training and were then tested by having each dog perform six searches in an odour-detection carousel with the trainer blinded to the location of the spore samples. In this high-stakes forced-choice test, each dog was asked to identify one new spore sample, containing approximately 93–265 million *P. larvae* spores, from seven control samples. Both dogs correctly identified the spore sample every time (100% success rate); the probability of this result occurring by chance was *p* = 0.0000038. Therefore, this study demonstrates that dogs can recognise an odour pattern from bacterial spore samples, in this case, *P. larvae*, and provides proof of concept for further investigation into the use of detection dogs to reduce the spread of AFB in beekeeping businesses.

## 1. Introduction

Detection dogs have long been utilised for their remarkable ability to detect a variety of chemicals, such as those present in explosives and illegal drugs [1,2]. More recently, dogs have been trained to identify odour patterns from a wide range of biological samples associated with different diseases, reviewed by Edwards et al. [3], and to identify particular species of animals, plants, and microorganisms [4]. Key to this ability is the recognition of particular combinations of volatile organic compounds (VOCs) emitted from biological products. VOCs are typically small molecules (< 300 daltons) with a lipophilic part, belonging to various chemical classes including alkenes, alcohols, ketones, benzenoids, pyrazines, sulphides, and terpenes [5]. Rather than detecting an individual VOC that is unique to a target, detection dogs are thought to recognise a specific pattern in the relative abundance of various types of VOCs emitted from a specific target, referred to here as the odour pattern.

In this study, we investigated whether trained detection dogs could recognise an odour pattern from spore samples of *Paenibacillus larvae* subsp. larvae, the causative agent of American Foulbrood (AFB) disease in honey bee (*Apis mellifera*) colonies. *Paenibacillus larvae* are Gram-positive rod-shaped bacteria that form spores which are highly resistant to heat and desiccation and can survive for many years in the environment [6]. Honey bee larvae become diseased by consuming infectious doses of *P. larvae* spores in their food. Following consumption, the spores germinate in the larval midgut and cause systemic bacteraemia that results in death at the prepupa or pupal stage, 9–12 days into development [7]. Large quantities of *P. larvae* spores persist in the dead larvae, with a single dead larvae containing around 2.8 × 10^9^ spores [8]. Removal of infected larvae by worker bees spreads the spores throughout the hive, infecting new larvae and contaminating the colonies honey stores [9]. Although some colonies appear to be able to limit or eliminate the disease, the majority of infected colonies rapidly succumb to the disease with the number of diseased larvae increasing exponentially over a period of weeks to months following the initial infection [10]. Spread of AFB disease occurs via robbing [11], where foraging bees from one colony enter and take honey from another colony that is infected with AFB [10], as well as beekeepers inadvertently introducing contaminated brood combs or feeding colonies with contaminated honey or pollen during routine management practises [11,12,13]. Regulations to limit the spread of AFB frequently require destruction of infected hives. Consequently, AFB is an economically important disease in many countries worldwide.

American Foulbrood disease is named after the characteristic sour or fishy odour emitted from heavily infected hives. A recent study has characterised the VOCs emitted from hives with clinical AFB, confirming that these hives have a unique VOC profile that differs in the abundance of different classes of VOCs, compared to healthy hives and those with other types of disease, and also contains a small number of VOCs unique to AFB [14]. Given the distinct odour pattern of AFB hives, it is unsurprising that there are numerous anecdotal reports of dogs being trained to recognise an odour pattern from AFB diseased larvae, and these dogs being used to detect AFB in the field. However, there are currently no studies published in peer-reviewed scientific journals, and results from government/industry reports in Australia and New Zealand have raised some concerns about false positive indications [15,16]. While these previous studies have focused on the detection of VOCs from diseased and decomposing larvae, our study investigated the ability of detection dogs to recognise an odour pattern from purified *P. larvae* spore samples. If detection dogs could identify hives and hive ware that is contaminated with spores, measures can be taken to prevent the spread of the spores before the clinical disease is apparent.

To date, several studies have investigated the use of detection dogs to recognise odour patterns associated with bacteria, such as *Staphylococcus aureus* and *Clostridioides difficile*, and found that dogs can readily identify these bacteria with diagnostic sensitivities of 83–100% [17,18,19]. While these studies investigated the detection of bacteria in pure culture [17,18], and samples from patients [18,19], another study investigated the detection of environmental reservoirs of *C. difficile* in a hospital setting [20]. Bryce et al. [20] trained a springer spaniel to recognise an odour pattern from pure *C. difficile* culture samples before progressing to simulated hospital searches, where the dog had a sensitivity of 80% and specificity of 93%. This dog was later successfully deployed in a hospital to identify areas that needed priority cleaning due to *C. difficile* contamination [20]. Detection dogs have also been successfully used in orchards to detect bacterial pathogens of citrus crops, with sensitivities in the range of 85.8–98.6% and specificities of 99.6–99.7% [21,22]. Training for these field operations started with an initial odour recognition phase, where the dogs were trained to identify the target odour pattern using as pure a sample as possible in a controlled set-up, before progressing to more complex environments [21,22].

Studies investigating dogs for the detection of bacterial pathogens have so far all used samples from vegetative bacteria [17,18,19,20,21,22]. To the authors’ knowledge, there are no peer-reviewed studies specifically investigating the detection of bacterial spores, which are metabolically inactive and thus expected to produce smaller odour patterns. In terms of structure, *P. larvae* spores are ellipsoidal, measuring about 0.6 × 1.3 µm [23], and comprised of a bilayered protein coat surrounding a peptidoglycan cortex and inner core [24]. The inner core contains cytoplasm, bacterial DNA and associated proteins, along with dormant ribosomes and enzymes. The spore core is highly mineralised, containing mainly Ca^2+^ ions chelated with dipicolinic acid which is important for reducing spore water content [6,25]. Direct detection of *P. larvae* spores by detection dogs would be ideal, given the central role of these spores in the transmission of the disease. However, although *P. larvae* spores are likely to emit some volatile compounds, the VOC concentration (vapour pressure) may be very low and it is currently unknown whether these spores would emit an odour pattern that could be recognised by trained detection dogs. Therefore, the aim of this study was to determine whether trained detection dogs can recognise an odour pattern associated with *P. larvae* spores. It is important to note that this study does not attempt to determine whether this odour pattern is specific for bacterial spores from this particular species. Rather, this study investigates the proof-of-concept that detection dogs can recognise an odour pattern from bacterial spores, in this case, *P. larvae* spores, in a highly controlled environment akin to the initial odour recognition phase that has been described in previous studies [20,21,22]. This study serves as a basis for future work to determine whether detection dogs could be a useful tool to reduce the spread of AFB in beekeeping businesses.

## 2. Materials and Methods

### 2.1. Sample Preparation

The initial strain of *Paenibacillus larvae* subsp. larvae was provided by the New Zealand Ministry of Primary Industries Animal Health Laboratory (MPI Reference number 7957), having originally been collected from a hive with clinically typical symptoms of American foulbrood in Auckland in May 2020. The strain was ERIC-I type. The method of *P. larvae* spore production was adapted from de Graaf et al. [26]. From the initial stock, 12 batches were produced each starting with 24 brain heart infusion (BHI) agar plates inoculated on a single day. The 12 batches were processed in parallel but kept separate so that 12 final spore samples were produced, each differing slightly in concentration and purity. An additional 12 uninoculated BHI agar plates were also processed alongside the *P. larvae* samples to serve as control samples. The plates were cultured for seven days at 37 °C, and the resulting bacterial lawns were collected by flooding with ice-cold deionized water and scraping the plates with a cell spreader [26]. Vegetative cells were devitalised and fragmented by holding samples at −20 °C for a minimum of 12 h followed by heating to 93 °C for 20 min. The spores were then purified and concentrated by four cycles of washing in reverse osmosis (RO) filtered water and centrifugation (12,000× *g*; 15 min; 4 °C). All steps were performed for both spore and control samples and the final samples were stored in RO-filtered water at 4 °C.

The quantity of *P. larvae* DNA in both spore and control samples was assessed with qPCR. Briefly, 200 µL of each sample was placed in a 2 mL screwcap tube containing a mixture of 0.5 mm and 2.3 mm zirconia silica beadbeating beads (BioSpec Products, Bartlesville, OK, USA), and homogenised in a Mini Beadbeater 16 instrument (BioSpec Products) for 3 min. DNA was extracted from the homogenised samples using the Bee Pathogen DNA/RNA Extraction Kit (Dnature Diagnostics & Research Ltd., Gisborne, New Zealand) according to the manufacturer’s instructions. Nucleic acid concentrations were measured with a Qubit 2.0 fluorometer. The extracted DNA was used as the template for qPCR, along with synthetic DNA standards, to calculate the *P. larvae* copy number. Quantitative PCR assays were run on a Mic qPCR Cycler (BioMolecular Systems, Gold Coast, Australia), using the commercially available AFB duo qPCR mastermix and primers (Dnature Diagnostics & Research Ltd.) and 2 µL of template DNA in a 10 µL reaction. This multiplex assay uses two *P. larvae* DNA targets: a single copy gene for quantification purposes (ftsZ, a prokaryotic homologue to tubulin) and the multicopy 16S gene [27]. The ftsZ probe is detected in the FAM channel and the 16S target in the HEX channel. The cycling conditions consisted of an initial 3-min denaturation step at 95 °C, followed by 45 cycles of 95 °C for 6 s and 60 °C for 20 s. Reactions were run in duplicate, and each plate included positive and negative controls and no template controls. Copies of *P. larvae* DNA per microliter template were determined from a standard curve generated from serial dilutions of the synthetic DNA standard. This measure was then used to calculate an estimated *P. larvae* spore concentration in the starting samples, with an estimated 50% DNA recovery during extraction and assuming that all *P. larvae* DNA was from the spores.

To assess sample purity, 10 µL from each spore and control sample was fixed on a glass microscope slide and stained with Schaeffer-Fulton’s method (Schaeffer and Fulton Spore Stain Kit, Sigma Aldrich, St. Louis, MO, USA). Stained slides were visualized microscopically at 1000× magnification.

Final spore and control samples, in 1.2–4 mL of RO filtered water, were sealed in 25 cm^2^ sterile, tissue-culture treated polystyrene culture flasks (Corning Inc., Corning, NY, USA) with 0.2 µm filter (vent) caps. The size of the filter caps allowed VOCs but not the spores to escape the flask [28]. To contain the volatile compounds, each flask was then stored in a 175 mL air-tight plastic container (Sistema Plastics, Auckland, New Zealand).

### 2.2. Dog Training

Three dogs were selected for this study, based on attributes identified during previous training for detection work. These attributes included a strong play drive, manifest as having a reward (toy) that they were motivated to receive, and a sustainable hunt drive meaning they were able to consistently search for a reward with their nose in the absence of visual cues. The dogs also had to be highly motivated to work with the trainer. The details of the dogs are shown in Table 1. They ranged in age from 2 to 3.5 years old and were Labrador retrievers or mixed (cross-bred) breeds. One was a spayed female, one a neutered male and one an entire (not-neutered) male. These dogs were owned and cared for by one of the authors (PG), a highly experienced trainer and handler of detection dogs.

**Figure 1:**
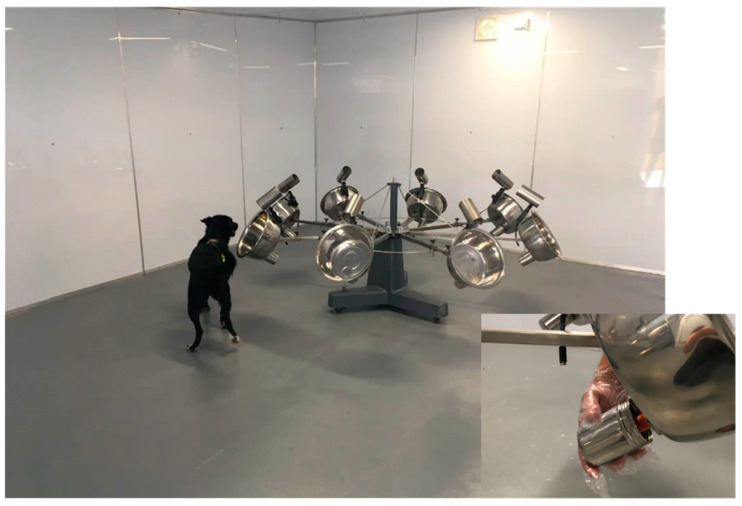
The testing carousel during training with Lillie. The insert lower right shows one of the ports with the sample pot disconnected and the orange cap of a control flask visible.

Prior to this study, all three dogs had undergone generic detection training following a similar method to that outlined for Customs Service narcotics detection dogs [29]. Training began by encouraging the dogs to detect a familiar toy (ball) by odour rather than sight. The dogs’ behaviour was then shaped over subsequent sessions, using positive reinforcement, to a consistent sit and forward focus once they had found the ball. An easily identified secondary odour, in this case a food spread, was then placed alongside the ball until the dogs learned to associate the two odour patterns. The ball was then removed, and only the secondary odour was hidden. A positive indication on the secondary odour was immediately rewarded with release of the ball. Eventually, the ball was replaced with an immediate verbal release, a high-pitched “yes”, that signalled an end to the search and was followed by a reward. The use of a secondary odour was standard practise for the dog trainer, as it allowed simulation of various narcotics detection search scenarios where the odour was concealed in a way that would not have been practical for a large item, such as a ball, and where the eventual target odour was difficult to detect or not consistently available. In this case, once the dogs were consistently indicating the secondary odour, they were then introduced to the odour-detection carousel. This was located in a purpose-built room at the training centre where the dogs resided. The carousel was a stainless steel structure on a rotatable base with eight arms, each with a port consisting of a large metal bowl with a grate in the bottom that communicated with a small compartment underneath, a stainless steel pot, that could hold a sample (Figure 1). The dogs were trained to enter the room and systematically sniff each of the bowls while the trainer waited at the entrance. A positive indication on the secondary odour concealed in one of the sample pots was released with a verbal “yes” and then rewarded with a ball throw outside of the testing room.

The dog training specifically for this study began with the introduction of the target odour, the *P. larvae* spore samples, alongside the secondary odour and then in isolation. To start with, one spore sample was introduced in place of the secondary odour in one port of the carousel, which was intentionally not cleaned to leave the residual VOCs from the secondary odour, while the other seven ports held polystyrene flasks containing saline that had previously been used as distractors during the generic training that pre-dated this study. Thus, the set-up was the same as the dogs had encountered during secondary odour training, except that instead of a flask containing the secondary odour, there was a spore sample associated with residual VOCs from the secondary odour. The spore sample used for this stage had the maximum number of spores available in the training set of samples. Each dog searched the carousel 0–3 times per day over five days, with the trainer rewarding changes in behaviour on the spore sample. After five days it was considered that the residual VOCs from the secondary odour would have dissipated and the dogs were indicating on the spore sample. To check this, the carousel was thoroughly cleaned to remove any residual VOCs from the secondary odour and reset with the spore sample and the same seven distractors (polystyrene flasks with saline). All three dogs continued to show a change in behaviour on the spore sample, ranging from a longer sniff of the spore sample (Ringo) to a clear sit (Wulf), suggesting they could recognise some difference between the spore and distractor samples. However, it was not known whether they were recognising an odour pattern from the spores, or some other difference between the spore samples and the known distractors (for example, only the spore sample flasks had labels).

Over the next eight days, the trainer varied the spore sample used, progressively using samples with a lower spore content until the dogs were working with the low-quantity training samples. The trainer also varied the amount of VOC dispersion time between loading the samples and the dog searches, and the order of the dogs, to check that these factors did not noticeably alter the dogs’ ability to find the target sample. The carousel was cleaned regularly. During all training, the trainer knew the correct location of the spore sample and ensured a positive outcome for the dog. Rather than letting a dog fail to find the spore sample, he shaped the dogs’ behaviour by adjusting the threshold for a successful search. For example, in the first few searches, he would release and reward the dog for simply pausing to sniff the spore sample, whereas the eventual aim was to have the dog sit and focus their attention on the correct port before being released and rewarded. This alteration in threshold was specific to the progress of each dog. If a dog was showing increasing confidence in their search the threshold would increase, whereas the introduction of more difficult conditions, for instance, a spore sample with a lower concentration of spores, was accompanied by a reduction in the threshold for a successful search. This method was used because the dogs had not yet convincingly recognised (imprinted) the target odour pattern. After 13 days (total) of target-odour training, two of the three dogs (Lillie and Wulf) were indicating on the spore sample with a clear sit and forward focus in all conditions, although it was still not known whether they were recognising an odour pattern from the spores, or some other difference between the spore samples and the known distractors. One dog, Ringo, had a faster and less thorough search pattern than the other two, and would inspect the port with the spore sample several times before giving a clear indication, but was nevertheless progressed to the next stage of training.

The next stage of training involved systematic desensitisation to all non-target odours in the spore sample. The previously used distractors were first replaced with new empty polystyrene flasks, and then new empty flasks with printed labels and sealed with Parafilm (Bemis, Neenah, WI, U.S.A.) and then new flasks containing RO purified water from the laboratory with labels and seals. This continued over six days and the dogs maintained their ability to indicate on the spore samples. The final control samples from the laboratory were then introduced as distractors. These control samples were processed from blank media in parallel with the spore samples, including all purification (washing) steps, and contained no *P. larvae* DNA as confirmed by qPCR. Thus, the final target odour pattern from training was comprised of the VOCs that were present in the spore samples but in not the laboratory-produced controls. These VOCs were from the *P. larvae* spores and any remaining debris from the vegetative bacteria that had persisted through the purification steps. All three dogs initially failed to indicate on the spore sample in the presence of the laboratory-produced control samples. The trainer then reverted to rewarding more subtle changes in behaviour on the spore sample. However, several days after the introduction of the final laboratory-produced control samples, which was 3 weeks into target odour training, Ringo was not showing any change in behaviour that could be shaped into an indication of the spore samples and his training was deemed unsuccessful. In contrast, Lillie and Wulf were showing some changes in behaviour at this time, such as spending longer sniffing the port with the spore sample, that suggested they could potentially differentiate the spore samples from the laboratory-produced control samples. The trainer continued training searches with these two dogs, using the spore samples and laboratory-produced control samples, and progressively increased the threshold for a successful search. Training was deemed successfully complete when both dogs clearly indicated, with a sit and forward focus, on the spore sample in training searches with one spore sample and seven laboratory-produced controls, in all three searches on a single day. Test trials were immediately scheduled and conducted shortly thereafter, leading to a total time from the start of target odour training to test trials of 5.5 weeks.

### 2.3. Dog Trials

Once the dogs were clearly indicating the target odour in training, they were then assessed in a series of six trials with new samples where the trainer was blinded to the location of the target odour. The dogs had not previously been exposed to blinded trials during training. The method of these trials can be seen in the video footage in supplementary materials (video S1). Prior to each run, the testing carousel was thoroughly cleaned; the sample pots were removed and immersed in boiling water for 3 min and the rest of the apparatus was wiped with 70% ethanol. Following cleaning, the carousel was left for 10 min for evaporation and dispersion of the ethanol. One researcher (NT) then set up the carousel with one spore sample and seven control samples from the testing set of samples that had been held in reserve. A new spore sample was randomly chosen for each run, along with seven controls that were randomly selected from a pool of 21 controls. Importantly, the dogs had never encountered these samples during training. When handling the samples, the researcher wore polyethylene gloves that the dogs were familiar with (used by the trainer), to avoid introducing human scent or another new odour onto the samples. The control samples were loaded first, starting at a random position determined using a random number generator on a Casio fx-82MS calculator at the time of the trial. Labels identifying the different ports during training had been covered and so position 1 was the port nearest the room entrance, with the numbering continuing in a clockwise direction. Gloves were changed before and after loading the spore sample. The researcher and videographer then left the testing room and waited in a nearby building so as not to inadvertently give body-language cues to the dogs. A tripod-mounted camera remained in the testing room to capture the dog work. After 10 min of odour dispersion, the trainer brought in the first dog. The researcher had previously allocated a random dog order for each trial and communicated this to the trainer. On entering the testing room, the dog would search by systematically inspecting the bowls until it found the target odour, which it would indicate with a sit and forward focus. The trainer then released the dog with a verbal high-pitched “yes” and rewarded the dog, even though the trainer did not know the location of the target odour, and marked the location of the indication by putting a plastic container on the floor under the bowl. Only a clear sit and forward focus was considered an indication by the dog. Once the dog and trainer had left the room, the researcher and videographer would re-enter and check the identity of the sample that the dog had indicated on. No container on the floor would mean that the dog had not indicated on any sample. The researcher would then disassemble the carousel and clean it, let the ethanol odour disperse, then load the carousel with the same set of samples, but starting in a different position, for the next dog.

The first trial of the six was used as a method check, where the trainer was told the outcome after the trial. Only one minor issue was identified which was one port that was not securely holding the sample pot. This required a minor adjustment in the method where, instead of spinning the carousel between trials, every time the samples were loaded a random number was generated which was used as the starting position to load the samples. This change had no impact on the dogs and so results from the initial trial were included in the analysis. Conditions were constant for the following five trials, and the trainer was not told the outcome of these until they had all been completed.

### 2.4. Results Analysis

The proportion of successful trials was analysed for each dog. Each trial represented a binomial test with a dichotomous outcome (successful or unsuccessful). A trial was deemed successful if the dog indicated on the correct sample, based on the trainer’s interpretation of where the dog indicated (the trainer was blinded to the correct location). No clear indication or indication of an incorrect sample was considered an unsuccessful trial. Using a binomial distribution for a dichotomous outcome test, with a probability of success of 1 out of 8, a total of five runs were required per dog so that the probability of a dog getting four or more successes by chance alone was *p* < 0.01. An extra run was included as a method check. This gave one opportunity to alter the testing conditions in the case of any unforeseen problems with the test design, in which case the first trial would be excluded from the analysis. Sensitivity (percentage of true positives correctly identified) and specificity (percentage of true negatives correctly identified) were not calculated, as the forced-choice trial design was not suitable for determining these measures [3].

## 3. Results

### 3.1. Samples

A total of 12 batches of *P. larvae* spores were produced with a mean concentration of 3.63 × 10^8^ copies of *P. larvae* DNA per mL and a range of 8.18 × 10^7^ to 1.37 × 10^9^ copies of *P. larvae* DNA per mL (Table 2). Half of the samples (A–F) were used for dog training; the approximate number of *P. larvae* spores in the training samples ranged from 2.7 billion in 4 mL, the maximum volume available for the initial training, down to 49 million in 1.2 mL in the later stages. The second half of the samples (H–M) were reserved for the dog trials and kept at a uniform volume of 1.2 mL, the maximum volume that was available for all samples, resulting in a mean spore number of 166 million, range of 93 to 265 million, spores per sample. This is approximately 6% of the number of spores that have been reported in a single dead larvae [8]. No *P. larvae* DNA was detected in the control samples produced in parallel from blank media nor in the negative and non-template controls used during DNA extraction and qPCR.

**Figure 2:**
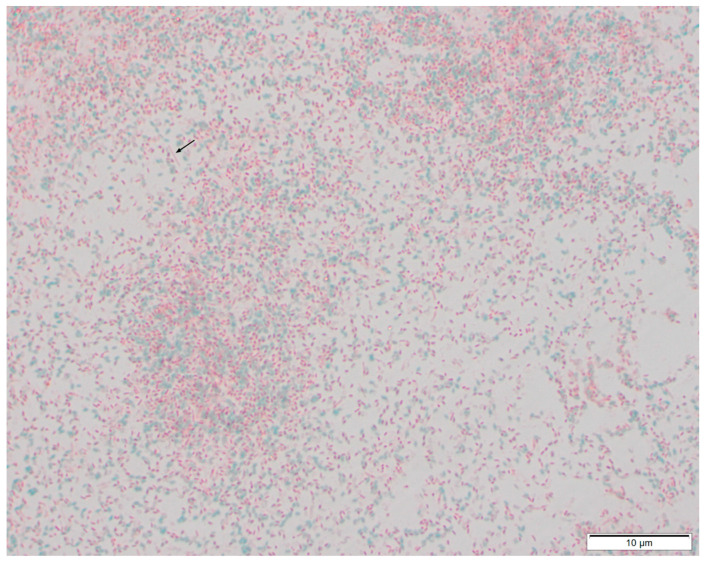
Photomicrograph of a representative high power field (1000× magnification) of sample M (no dilution) containing 3.33 × 10^8^ copies of *P. larvae* DNA per mL corresponding to approximately 200 million spores in the 1.2 mL sample. Stained with Schaeffer-Fulton’s method; the numerous green ovoid structures are free spores, while the smaller magenta bodies are spores in the late stages of development within *P. larvae* mother cells. A vegetative *P. larvae* bacterium is shown with an arrow.

Inspection of the samples under light microscopy (1000× magnification) with Schaeffer-Fulton staining showed that all samples contained large numbers of both free spores and spores still contained within *P. larvae* mother cells in the late stages of development (Figure 2). In most cases, only small numbers of vegetative cells could be seen (samples D, E, F, H, I, J, K, L, M), amounting to less than 5% of the sample. However, some samples had numerous vegetative bacteria (samples A, B, C), in approximately equal proportions to the spores. It appears that the purification had been ineffective in these samples and so these samples were only used for dog training and not for the final trials.

### 3.2. Dog Trials

The two trained dogs (Lillie and Wulf) were each assessed in six trials with unused samples. Each trial involved one search of the carousel by each dog and the trainer was blinded to the location of the spore sample. In each of the six trials, both dogs correctly indicated on the spore sample (Table 3), corresponding to a 100% success rate for both dogs. There were no false positive indications. For each dog, the probability of this result being due to chance alone was *p* = 0.0000038. Video footage compiled from trials 5 and 6 is included (video S1), and footage from all of the trials is available on request.

The two dogs used different search patterns; Lillie systematically sniffed each port, working in a clockwise direction. In contrast, Wulf worked in an anti-clockwise direction, first completing a fast search of the carousel before honing in on individual ports. The searches took between 11 and 43 s, with a mean of 23.8 s (95% CI: 18.3–29.3). Two of the searches were <15 s, with the dog clearly indicating on a port at the first sniff. For an example, see trial 6 dog 1 in the accompanying video (video S1). The rest of the searches took 15–45 s and involved an initial change in behaviour at a port, but not a sit and forward focus, in which case the trainer would direct the dog to keep searching. In some cases this would lead to a clear indication on a different port (e.g., trial 5, dog 2). Other times the dog would return to the same port and give a clear indication (e.g., run 5, dog 1).

## 4. Discussion

The results of this study provide proof of concept that dogs can recognise an odour pattern from *Paenibacillus larvae* spore samples; two out of three dogs successfully completed training and were consistently able to detect laboratory-produced spore samples in an odour-detection carousel. To the authors’ knowledge, this is the first peer-reviewed study demonstrating that dogs can recognise an odour pattern from bacterial spores, in this case, *P. larvae* spores, although the odour pattern detected may not be specific for this species of bacteria. This study represents an early step towards the development of *P. larvae* spore detection dogs as a tool for reducing the spread of American Foulbrood (AFB) disease in beekeeping businesses.

While there are no previous studies on the use of detection dogs for *P. larvae* spores, there are two government/industry reports on the use of dogs to detect dead or diseased honey bee larvae from hives with AFB [15,16]. In the large field trial, an AFB detection dog was run past approximately 2000 hives on apiaries in an area where AFB was present [15]. The dog indicated on 77 hives, but only 7 of these were confirmed to have AFB by qPCR testing. Not all of the 2000 hives were tested and the report had limited information on the dog work as this was not the primary focus. However, an interesting observation from the authors was that, in the positively indicated hives, the colonies often appeared to be in poor health for a range of reasons but for nearly all hives this was not because of AFB. Therefore, the false positive indications may be because the dog was trained on material taken from hives with clinical AFB, which would have a large and complex odour pattern including volatile organic compounds (VOCs) that may be increased in many conditions of stress or disease that are not specific to AFB. The second report found that a Labrador Retriever presented with samples of muslin cloth that had been stored in 51 hives, including four with clinical AFB, correctly indicated on one of the AFB samples and gave partial indications on the other three samples from the AFB hives [16]. The dog also indicated on two healthy hives and gave partial indications on a further 12 healthy hives. Although there were limited details on the training methods used, the authors did comment that training was only successful after the introduction of pure samples of honeybee larvae that had been infected with *P. larvae* in a laboratory, rather than samples taken from clinically infected hives. Both reports also noted the challenge of protecting dogs from bee stings while they were working.

These early reports suggest that detection dogs may have potential as a screening test for AFB on apiaries, where positive indications are followed up with visual inspection and qPCR. However, the training method used, particularly the availability of laboratory-produced control samples, will likely impact the dogs’ performance. The main advantage of using dogs as an initial screening test is that they can quickly work through a large number of hives spread over a wide area, facilitating more thorough inspections than would be possible by individually assessing or sampling hives. Additionally, suspect hives can be identified at the time and further investigated with visual assessment, rather than needing to revisit the apiary after the results of molecular testing. However, further work is needed to better understand the capabilities and limitations of AFB detection dogs and to compare this with visual inspections, as well as other screening methods, such as qPCR from pooled samples of honey or bees, or the recently described Foster method (qPCR of hive entrance swabs) [27].

In this study, we focussed on *P. larvae* spores because of their central role in the transmission of the disease and thus the potential for mitigating the spread of AFB. Firstly, the use of detection dogs to identify hives with high levels of *P. larvae* spores that do not display clinical symptoms may enable beekeepers to quarantine these hives and later destroy those that display clinical symptoms. Secondly, honey supers, the upper part of the hive that collects honey, do not typically contain dead larvae and so dogs that were able to detect *P. larvae* spores could be used to screen honey supers that are stored over winter or during seasons when the honey flows are scarce. With further research, honey supers that are heavily contaminated with spores could possibly be identified and either quarantined or destroyed before the supers are added the following season. This may also be the case for detecting *P. larvae* spores in empty brood boxes from colonies that have died for other reasons. Detection in both cases would prevent the introduction of *P. larvae* to healthy hives with the further advantage that the dog work could be performed away from the hives with less risk of the dogs getting stung.

Laboratory-produced spores were used in this study, rather than dead honey bee larvae, so that the dogs could be trained to identify an odour pattern from the spores themselves. Large numbers of spores (approximately 49 million to 2.7 billion) were included in each sample in this study because the odour pattern was expected to be small given the spores are not metabolically active. These quantities are approaching what would be expected in a single dead larvae (2.8 billion) [8]. The requirement for laboratory-produced spore samples with high concentrations limited the number of samples that were available for this study. Additionally, separate sample sets were used for training and testing to ensure that the dogs were identifying a common odour pattern rather than memorizing the odour pattern of individual samples that were correct during training [30]. Given the limitations on sample size, the most appropriate test design was a forced-choice test, where the dogs were asked to identify one spore sample from seven controls in a carousel, with new samples and only six attempts. This is a high-stakes scenario where even a small number of false indications could have a large effect on the overall result. Therefore, the high success rate of two dogs in this study provides convincing evidence that dogs can recognise an odour pattern from *P. larvae* spore samples, but the test design was not suitable for evaluating parameters such as sensitivity and specificity [3]. These would require additional repetitions, requiring a large number of samples, in an operational (field) setting where there may be multiple positive samples or no positive samples in a single search [3].

Another limitation of this study was the use of spores from only a single bacterial species, in this case, *P. larvae*. Many of the VOCs emitted from these spores may have come from the spore coat, or other structural components that are common to bacterial spores from other species, including those present in the environment [31]. Therefore, the odour pattern that the dogs recognised in this study may not be specific for *P. larvae* and further work is needed to investigate whether detection dogs can discriminate between the *P. larvae* spores and spores from other types of bacteria that may be present in working hives and on hive ware in storage.

The intended target odour in this study was *P. larvae* spores and the dogs were systematically desensitised to other known non-target odours in the spore samples, such as the polystyrene flasks and purified water. However, microscopic inspection of Schaeffer-Fulton stained spore samples showed that all target odour samples contained both free spores and spores still contained within *P. larvae* mother cells in the late stages of development. Thus, it is possible that the remnants of the mother cells were also contributing VOCs to the odour pattern. In a field setting, hives are likely to contain both free spores and those still developing in mother cells, but other samples, such as honey supers would be expected to contain predominantly free spores. Further dog training may be required if the target odour pattern in the field is slightly different from that of the laboratory-produced spores. Furthermore, only a single strain of *P. larvae* was used in the current study, and different strains may also present slightly different odour patterns.

The progression from training to testing in this study was based on the dogs showing a clear indication, comprised of a sit and forward focus, on the spore sample in the training searches with the same set-up as the trials (one spore sample and seven laboratory-produced control samples that had been processed in parallel) in all searches on a single day. This was a subjective criteria that relied on the trainer’s interpretation of a clear sit and forward focus. This contrasts to a recent study describing a quantitative criteria to determine the transition to accuracy testing [32]. Such criteria were not possible in this study because the training method did not involve unsuccessful searches, using instead a progressively increasing threshold for a successful search. This method was used because the dogs had not yet recognised (imprinted) the target odour and so unsuccessful searches might comprise their understanding of the target odour pattern. However, this study represents an early proof of concept of odour recognition and we did not attempt accuracy testing. Further studies investigating accuracy measures, such as sensitivity and specificity, should be conducted in the field and use a standardised criteria for progression to testing to facilitate comparison with other studies.

One of the dogs in this study did not complete target odour training because he did not appear to be able to distinguish the spore samples from the control samples that were produced in parallel from blank media. The trainer reported that this dog had a less mature search pattern than the other two dogs in that he would sniff as he moved around the carousel but he did not systematically put his nose in each bowl. In the trainer’s experience, this type of search pattern is sufficient to detect large odour patterns with high concentrations of VOCs, but not small odour patterns. This is an anecdotal observation from the trainer which supports our hypothesis that *P. larvae* spores have a small odour pattern based on their lack of metabolic activity. In a field situation, there would likely be high numbers of *P. larvae* spores in infected hives and more time for VOCs to accumulate [8]. However, there will also be other strong odours that may mask the odour pattern from *P. larvae* spores. In contrast, hive ware such as honey supers or empty brood boxes would be expected to have fewer spores but also fewer additional odours. Therefore, the performance of the dogs in this study, with purified spore samples in a detection carousel, may not relate to how these dogs would perform in the field and further work is needed to evaluate dog performance for each specific field application.

Interestingly, all of the dogs initially struggled to identify the spore samples when the laboratory-produced controls were introduced, despite previously being able to consistently distinguish between the spore samples and identical flasks containing purified water from the laboratory. This suggests that there was a non-target odour in both the laboratory-produced spore and control samples, which was not from the flask, label, seal or purified water, and was contributing significantly to the odour pattern. We hypothesize that this compound(s) was either remnant from the media, despite four washing steps, or it was from the plastic ware used in the preparation of these samples. This unidentified compound seems to have contributed more to the odour pattern than the millions of *P. larvae* spores in the spore samples. The fact that two of the dogs still managed to detect some difference between the laboratory-produced spore and control samples, and were able to consistently indicate on the spore samples with some further training, demonstrates that there was a recognisable target odour from the *P. larvae* spores and remnant mother cells. Therefore, despite the use of laboratory-produced purified samples, we still had non-target odours in our training sample, akin to the non-target odours from material taken from AFB hives [15]. However, the use of laboratory-produced parallel control samples in this study allowed us to identify the presence of non-target odours during training and correct for these with further desensitisation. The inclusion of non-target odours in positive training samples is a known problem in detection dog training [33], which can be controlled by desensitisation or by using a wide range of positive samples [3]. However, it is not always possible to control for all non-target odours in positive training samples. Often this has little practical significance, for example, if the non-target odour is a small component of the odour pattern, or if the target and non-target odours always occur together. However, problems can arise in the field if there is a low incidence of the odour pattern that the dog is trying to find. In absence of the target odour, the dog can be tempted to indicate a similar odour pattern that contains some of the same (non-target) odours, but without the target odour leading to a false positive indication [33]. This illustrates the importance of carefully designed training in detection dog work.

## 5. Conclusions

This study demonstrates that trained detection dogs can recognise an odour pattern from *Paenibacillus larvae* spore samples. Further work is needed to investigate the accuracy (sensitivity and specificity) and feasibility of using detection dogs in an operational setting to determine whether they could be a useful tool to reduce the spread of American Foulbrood in beekeeping businesses.

## Figures and Tables

**Table 1:** Details of the dogs used in this study, s: spayed, e: entire (not neutered), n: neutered. Heading dog is a NZ sheep herding dog breed derived from Border Collies.

Name	Breed	Age (Years)	Sex	Previous Experience
Lillie	Labrador cross	2	F (s)	Demonstration dog for detection work
Wulf	Heading dog cross	3	M (e)	Trained for border security (customs) work
Ringo	Labrador retriever	3.5	M (n)	Trained for border security (customs) work

**Table 2:** Concentration of *P. larvae* DNA and approximate spore numbers in the training and testing samples. Spore samples A–F were used in training, with the same sample used at several volumes, e.g., sample A was first used at 4 mL (A1) and then reduced to 1.2 mL (A2). Four control samples (N–Q) were divided into 7 controls for training. Spore samples H–M were used in the trials, along with control samples R–Y which were divided into 21 controls at 1.2 mL each. −: none detected by qPCR. ^1^ copies of *P. larvae* DNA per mL, ^2^ See methods for calculation of approximate number of *P. larvae* spores per sample.

Training					Testing				
Lab Sample	*P. larvae* DNA ^1^	Sample Name	mL	Spores ^2^ (million)	Lab Sample	*P. larvae* DNA	Sample Name	mL	Spores (million)
A	1.37 × 10^9^	A1 A2 A3	4 1.2 0.2	2740 822 137	H	2.29 × 10^8^	H	1.2	137
B	4.26 × 10^8^	B1 B2	4 1.2	852 256	I	3.30 × 10^8^	I	1.2	198
C	5.24 × 10^8^	C1 C2	4 1.2	1048 314	J	4.42 × 10^8^	J	1.2	265
D	8.18 × 10^7^	D1 D2	4 1.2	164 49	K	1.55 × 10^8^	K	1.2	93
E	2.03 × 10^8^	E1 E2	4 1.2	406 122	L	1.68 × 10^8^	L	1.2	101
F	1.90 × 10^8^	F1 F2 G1 G2	2 1.2 2 1.2	190 114 190 114	M	3.33 × 10^8^	M	1.2	200
N	−	2.1 2.2	4 1.2	− −	R	−	1a 1b 1c	1.2 1.2 1.2	− − −
O	−	1 3	1.2 1.2	− −	S	−	2a 2b 2c	1.2 1.2 1.2	− − −
P	−	4 5	1.2 1.2	− −	T	−	3a 3b 3c	1.2 1.2 1.2	− − −
Q	−	6 7	1.2 1.2	− −	U	−	4a 4b 4c	1.2 1.2 1.2	− − −
					V	−	5a 5b 5c	1.2 1.2 1.2	− − −
					X	−	6a 6b 6c	1.2 1.2 1.2	− − −
					Y	−	7a 7b 7c	1.2 1.2 1.2	− − −

**Table 3:** Dog indications on *P. larvae* spore samples conducted in six blind trials using two trained dogs. Dog 1: Wulf, dog 2: Lillie. ^1^ Approximate quantity of *P. larvae* spores (millions) in the 1.2 mL sample; see methods for calculation. ^2^ Refers to the position (port) in the odour-detection carousel. ^3^ Trial 2 dog 2 search time is approximate due to a technical issue with the room camera.

	Spore Sample (Quantity ^1^)	Control Samples	Position ^2^ of Spore Sample	Dog 1 Position Indicated	Dog 2 Position Indicated	Search Time (Seconds)
Trial 1	I (198 m)	1a 1b 1c 2c 4a 5a 6c	6	6		30
			7		7	17
Trial 2	K (93 m)	1a 1b 2a 2b 4c 5a 7c	2	2		31
			4		4	30 ^3^
Trial 3	L (101 m)	2a 2b 3c 4c 5b 6b 7a	6	6		15
			1		1	34
Trial 4	J (265 m)	1c 2c 3a 5b 5c 6b 6c	4	4		43
			6		6	11
Trial 5	H (137 m)	3a 3b 4b 5c 6a 7b 7c	4		4	20
			5	5		22
Trial 6	M (200 m)	3b 3c 4a 4b 6a 7a 7b	8		8	20
			1	1		13

## Data Availability

The data presented in this study are available on request from the corresponding author.

## Supplementary Materials

The following supporting information can be downloaded at: https://zenodo.org/record/7223159#.Y1tJhGf7RPa, Video S1: Dog trials 5 & 6 (published 19 October 2022).
